# Composites of Laponite and Cu–Mn Hopcalite-Related Mixed Oxides Prepared from Inverse Microemulsions as Catalysts for Total Oxidation of Toluene

**DOI:** 10.3390/ma11081365

**Published:** 2018-08-06

**Authors:** Bogna D. Napruszewska, Alicja Michalik, Anna Walczyk, Dorota Duraczyńska, Roman Dula, Wojciech Rojek, Lidia Lityńska-Dobrzyńska, Krzysztof Bahranowski, Ewa M. Serwicka

**Affiliations:** 1 Jerzy Haber Institute of Catalysis and Surface Chemistry, Niezapominajek 8, 30-239 Krakow, Poland; ncnaprus@cyf-kr.edu.pl (B.D.N.); ncmichal@cyf-kr.edu.pl (A.M.); ncawalcz@cyf-kr.edu.pl (A.W.); ncduracz@cyf-kr.edu.pl (D.D.); ncdula@cyf-kr.edu.pl (R.D.); ncrojek@cyf-kr.edu.pl (W.R.); 2 Institute of Metallurgy and Materials Science, Polish Academy of Sciences, Reymonta 25, 30-059 Krakow, Poland; l.litynska@imim.pl; 3 Faculty of Geology, Geophysics and Environmental Protection, AGH University of Science and Technology, al. Mickiewicza 30, 30-059 Krakow, Poland; bahr@agh.edu.pl

**Keywords:** Laponite/hydrotalcite composite, organoclay, inverse microemulsion, Cu–Mn–Al mixed oxides, combustion catalysts, Zr dopant, Ce dopant

## Abstract

Composites of Laponite and Cu–Mn hopcalite-related mixed oxides, prepared from hydrotalcite-like (Htlc) precursors obtained in inverse microemulsions, were synthesized and characterized with XRF, XRD, SEM, TEM, H_2_ temperature-programmed reduction (TPR), and N_2_ adsorption/desorption at −196 °C. The Htlc precursors were precipitated either with NaOH or tetrabutylammonium hydroxide (TBAOH). Al was used as an element facilitating Htlc structure formation, and Ce and/or Zr were added as promoters. The composites calcined at 600 °C are mesoporous structures with similar textural characteristics. The copper–manganite spinel phases formed from the TBAOH-precipitated precursors are less crystalline and more susceptible to reduction than the counterparts obtained from the precursors synthesized with NaOH. The Cu–Mn-based composites are active in the combustion of toluene, and their performance improves further upon the addition of promoters in the following order: Ce < Zr < Zr + Ce. The composites whose active phases are prepared with TBAOH are more active than their counterparts obtained with the use of the precursors precipitated with NaOH, due to the better reducibility of the less crystalline mixed oxide active phase.

## 1. Introduction

The emission of volatile organic compounds (VOCs), stemming mainly from industrial processes and automobile exhausts, represents a serious environmental hazard, particularly in developed countries. Catalytic combustion is considered a particularly attractive clean-up technology for VOC mitigation. The catalysts for this process are based either on noble metals or on transition metal oxides [1,2,3,4,5]. The latter are considerably cheaper and, frequently, more thermally stable and less susceptible to poisoning. The Cu–Mn–O system has a special place in the history of total oxidation catalysis. During World War I, the search for an efficient CO absorbent to be used in military gas masks resulted in the development of hopcalite catalysts—based on mixtures of Cu and Mn oxides—which are extremely efficient in CO oxidation [6,7]. Research into catalytic applications of the Cu–Mn–O system has been continued ever since, especially given that the materials show high activity in the total oxidation of VOCs [8,9,10,11,12,13,14,15,16,17,18,19,20]. In several cases, hydrotalcite (Htlc)-like precursors, which, when thermally decomposed, yield mixed oxides of exceptional properties, were used [9,14,15,18]. However, the combustion catalysts based on transition metal oxides are prone to thermal deterioration due to sintering phenomena, enhanced by the strongly exothermic reaction environment [21]. In order to counter this effect, we recently put forward a new idea for the design of VOC combustion catalysts, based on the combination of Htlc nanoparticles obtained by an inverse microemulsion method with the exfoliated organosmectite (Laponite or montmorillonite) [22,23,24]. After calcination, such composites contain nanograins of the active mixed oxide phase held apart by the chemically inert silicate layers, which renders the catalyst more resistant to deactivation. The to-date investigated catalytic systems have been based on Mn as the active transition metal component, with Al present as a hydrotalcite structure-forming element. The research has addressed the effect of the Htlc phase loading [22], the role of promoters such as Ce and Zr [24], and the buildup of a hierarchical structure by entrapment of TiO_2_ nanoparticles prior to MnAl Htlc ones [23].

In the present work, we decided to extend our study to the testing of the mixed oxide active phase related to hopcalite formulae. Laponite was used as the clay component and Cu–Mn–Al Htlc as the active phase precursor. In such a system, the synthetic work faces complications, because the inverse microemulsion of NH_3_ aq, routinely used as a precipitating agent to be combined with the microemulsion of metal salts, is not suitable in the case of Cu-containing precursors, due to the ease of formation of soluble copper (II) ammonia complexes. Therefore, the syntheses of the hydrotalcite precursors in inverse micelles were carried out with two alternative precipitating agents: sodium hydroxide and, for the first time, tetrabutylammonium hydroxide (TBAOH). It has been reported that the use of a tetraalkylammonium hydroxide for the synthesis of metal hydroxide precursors enables the formation of nanocrystalline oxide particles with unique properties [25]. The obtained composites were tested in the combustion of toluene.

## 2. Materials and Methods

### 2.1. Materials

Htlc nanoparticles of the active phase precursors were prepared using the modified inverse microemulsion method [26], employed by us previously to synthesize Mn–Al Htlc micellar precursors with a Mn/Al ratio equal to 4:1.3 (i.e., ≈3) [22,23,24]. In order to achieve the hopcalite-like composition of the active phase, 1/3 of Mn has been replaced with Cu, so the nominal atomic Cu/Mn/Al ratio was set at 1.3:2.7:1.3. The major change in the synthetic procedure consisted of the use of aqueous solutions of NaOH or TBAOH, rather than NH_3aq_, as precipitants. An inverse microemulsion may be described as an isotropic and thermodynamically stable mixture of aqueous phase, hydrocarbon (oil), and surfactants, in which micelles with aqueous cores are dispersed in the oil phase [27]. In this work, the organic medium was composed of isooctane as the oil phase, cetyltrimethylammonium bromide (CTABr) as a surfactant, and n-butanol as a co-surfactant in weight proportions of 2:1:1.5, respectively. The inverse microemulsion reagent containing the Htlc-forming metal elements was prepared by dispersing an aqueous solution of Cu(NO_3_)_2_, Mn(NO_3_)_2_, and Al(NO_3_)_3_ (concentration 0.13 M, 0.27 M, and 0.13 M, respectively) in the organic medium (10 mL aqueous phase: 42.4 g organic phase), until an optically transparent liquid was obtained. The precipitating reagents were obtained by dispersing an aqueous NaOH solution (concentration 1.0 M) or aqueous TBAOH solution (concentration 0.8 M) in the organic medium (10 mL aqueous phase: 42.4 g organic phase). The dispersion of NaOH solution in the organic medium yielded macro rather than microemulsion, as indicated by the slightly turbid appearance of the liquid. In the case of TBAOH, a clear microemulsion was formed. Equal volumes of microemulsion with metal salts and microemulsion (macroemulsion) with the precipitating base were mixed and aged at 70 °C for 16 h. Zr and Ce promoters (5:1 atomic ratio) were introduced as ZrO(NO_3_)_2_ and Ce(NO_3_)_3_ to the inorganic salts solution. The dopants replaced part of Al. The following samples were obtained: Cu_1.3_Mn_2.7_Al_1.3_(im-NaOH), Cu_1.3_Mn_2.7_Ce_0.1_Al_1.2_(im-NaOH), Cu_1.3_Mn_2.7_Zr_0.5_Al_0.8_(im-NaOH), and Cu_1.3_Mn_2.7_Zr_0.5_Ce_0.1_Al_0.7_(im-NaOH), for materials precipitated with NaOH; and Cu_1.3_Mn_2.7_Al_1.3_(im-TBAOH), Cu_1.3_Mn_2.7_Ce_0.1_Al_1.2_(im-TBAOH), Cu_1.3_Mn_2.7_Zr_0.5_Al_0.8_(im-TBAOH), and Cu_1.3_Mn_2.7_Zr_0.5_Ce_0.1_Al_0.7_(im-TBAOH), for the TBAOH precipitated series, with subscripts indicating the intended metal elements ratio. The suspensions of the (im) precursors were used for the preparation of the composites. For the purposes of comparison, a Cu–Mn–Al Htlc was prepared by the conventional co-precipitation at pH = 10, by adding solution of appropriate metal nitrates to a solution of sodium carbonate, with the pH maintained by the addition of 1 M of NaOH. This reference is denoted as Cu_1.3_Mn_2.7_Al_1.3_(st-NaOH).

Laponite RD (Rockwood Additives Ltd., Cheshire, UK), a synthetic smectite with an empirical formula of Na_0.7_[Si_8_Mg_5.5_Li_0.3_O_20_(OH)_4_] (Na-L) was transformed into an organoclay by cation exchange with cetyltrimethylammonium (CTA) cations in an aqueous CTABr solution. The isopropanol dispersion of organoclay (CTA-L) was mixed with the Htlc (im) precursor suspension, to obtain ca. 1:3 active phase/clay wt. ratio. After aging for 2 h at 20 °C, the catalyst precursors were separated by filtration, subjected to lyophilization, and calcined at 600 °C (6 h). The severe conditions of calcination were chosen as a means of ensuring possibly high resilience of the catalysts against thermal deterioration during catalytic reaction. The general samples signature is M(1)M(2)…(im_base)/CTA-L, where M(1), M(2), and so on refer to the metal elements in the active phase, and base stands for the NaOH or TBAOH precipitation route. In addition, a reference composite was prepared, with the active phase of the same composition as in the case of the best catalyst prepared by inverse microemulsion but obtained by the standard co-precipitation, with the clay component being the parent Laponite (Na-L). The aqueous suspensions of both components were mixed, and the solid filtered, lyophilized, and calcined as with other catalysts. The reference catalyst is denoted as CuMnAlZrCe(st-NaOH)/Na-L.

### 2.2. Methods

X-ray diffraction (XRD) was studied using a X’Pert PRO MPD diffractometer (PANalytical, Almelo, the Netherlands), Cu Kα radiation (40 kV, 30 mA), a nickel monochromator, and a step size of 0.05°/min.

Chemical analysis of the catalysts was carried out with a ZSX Primus II (Rigaku, Tokyo, Japan) spectrometer (Rh anode) using a calibration based on the certified reference materials.

High-magnification SEM micrographs of uncoated samples were obtained with a JSM-7500F (JEOL, Tokyo, Japan) field emission scanning electron microscope (SEM).

Transmission electron microscopic (TEM) studies were performed using a Tecnai G2 (FEI, Eindhoven, the Netherlands) high-resolution transmission electron microscope, at 200 kV, equipped with a high-angle annular dark field scanning transmission microscopy detector (HAADF-STEM) combined with an energy dispersive X-ray (EDX) EDAX microanalysis.

Temperature-programmed reduction (TPR) was carried out in a quartz flow reactor, using ca. 0.015 g of the sample, 5 vol.% H_2_ in Ar (Linde, H_2_ 5% in Ar at flow rate of 30 mL/min), and a thermal conductivity detector (TCD). A temperature ramp of 10 °C/min in the range from room temperature to 700 °C was used. Fityk 0.9.8 software was used for deconvolution of the TPR profiles [28].

N_2_ adsorption/desorption at −196 °C was measured with an AUTOSORB 1 (Quantachrome, Boynton Beach, FL, USA) instrument. The samples were outgassed at 200 °C for 3 h. BET formalism was used for the calculation of specific surface areas, and t-plot was used for the micropore surface area (S_micro_) and micropore volume (V_micro_) evaluation. The total pore volume (V_tot_) was determined from the amount of N_2_ adsorbed at p/p_0_ = 0.996. The mean pore diameter (D^av^) was calculated with the D^av^ = 4V_tot_/S_BET_ formula.

The catalytic combustion of toluene was conducted in a fixed-bed flow quartz reactor, using ca. 0.5 g of a catalyst (0.3–0.5 mm fraction) in the temperature range of 100–400 °C. A mixture of 500 ppm toluene in air was fed to the catalyst at GHSV of 10,000 h^−1^. The reaction products were CO_2_ and H_2_O. The toluene was analyzed with a GC-FID (SRI 8610A) and CO_2_ with a GC-FID (SRI 310).

## 3. Results and Discussion

### 3.1. Characterization of Composites

**Figure 1:**
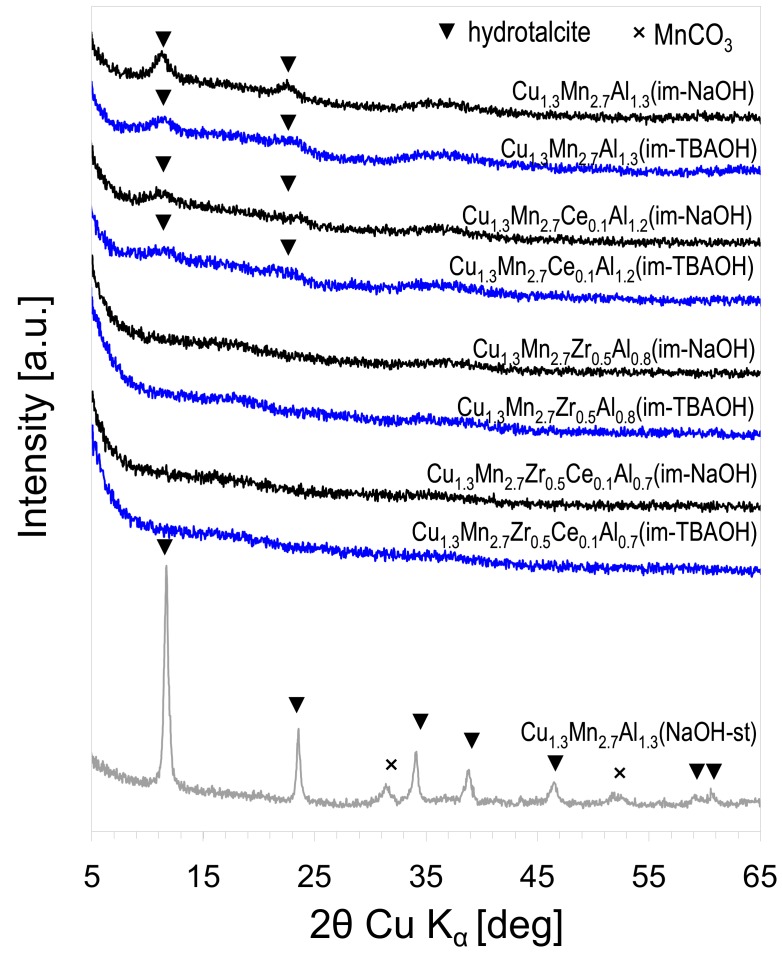
XRD patterns of mixed oxide precursors obtained by the inverse micellar route. XRD patterns of selected precursors obtained by the standard co-precipitation are shown for comparison.

XRD diagrams of active phase precursors obtained by the inverse micellar route are shown in Figure 1. For comparison, the XRD patterns of Cu_1.3_Mn_2.7_Al_1.3_(st-NaOH) prepared by the standard co-precipitation method are also included. The reference material showed intense reflections characteristic of a Htlc lattice (JCPDS file 00-051-1526). Additionally, reflections pointing to the presence of some MnCO_3_ impurity (JCPDS file 04-001-7250) were visible, which is frequent in Mn-containing Htlc synthesized by co-precipitation in the presence of carbonate [29]. The active phase precursors precipitated within the micelle volume were much less crystalline, and only those without the addition of Zr (i.e., Cu_1.3_Mn_2.7_Al_1.3_(im-NaOH), Cu_1.3_Mn_2.7_Ce_0.1_Al_1.2_(im-NaOH), Cu_1.3_Mn_2.7_Al_1.3_(im-TBAOH), and Cu_1.3_Mn_2.7_Ce_0.1_Al_1.2_(im-TBAOH)) showed traces of 003 and 006 Htlc reflections (barely noticeable in the materials containing cerium). The loss of crystallinity was attributed, on the one hand, to the dimensional restrictions exerted by the micelles and, on the other, to the high concentration of reactants used in this synthesis, favoring the formation of multiple crystallization seeds. The precursors containing the Zr additive were completely amorphous. The poorer resolution of the Htlc XRD patterns in the samples doped with Zr and Ce has been observed before [24]. The effect is taken as an indication that the dopants enter, at least partly, the Htlc structure and, due to the incompatibility of Ce^3+^’s large ionic radius (1.01 Å) or the charge of Zr^4+^ with the Htlc structure, spoil the long-range order in the precipitated solid. In addition, it was observed that the change of the precipitating agent from NaOH to TBAOH yielded even fewer ordered Htlc structures.

**Figure 2:**
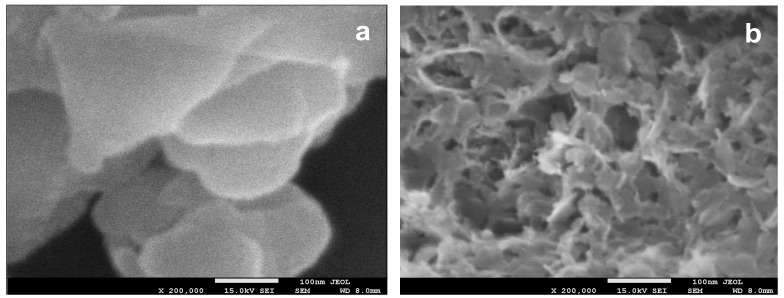
SEM images of: (**a**) Cu_1.3_Mn_2.7_Al_1.3_(st-NaOH); and (**b**) Cu_1.3_Mn_2.7_Al_1.3_(im-NaOH); (uncoated specimens). The white bars correspond to 100 nm.

Preparation of the active phase precursors within the volume of inverse micelles was shown to produce much smaller particles than the conventional co-precipitation [22,24]. A similar effect was observed in the case of the Cu–Mn–Al based system studied in this work. As an example, Figure 2 enables the comparison of a high-magnification SEM image of the reference Cu_1.3_Mn_2.7_Al_1.3_(st-NaOH) sample (Figure 2a) with the image of the Cu_1.3_Mn_2.7_Al_1.3_(im-NaOH) precipitate obtained by the inverse microemulsion method (Figure 2b). It may be seen that the latter is composed of very fine, platy particles, which are much smaller than the plate-like grains visible in the sample obtained by the standard co-precipitation method. This shows the impact of spatial limitations, which are imposed on the material formed within micelles but are absent in the conventional Htlc synthesis. The fine grain morphology is typical of all active phase precursors obtained by the inverse microemulsion method.

The results of the XRF analysis of the investigated composite samples are presented in Table 1. The ratios of key redox elements to the aluminum component are close to the nominal values, indicated by the formulae of particular active phases. The reference sample contains some sodium, due to the use of Na-L as the clay component, while all the materials prepared with the CTA-L organoclay are Na-free.

**Figure 3:**
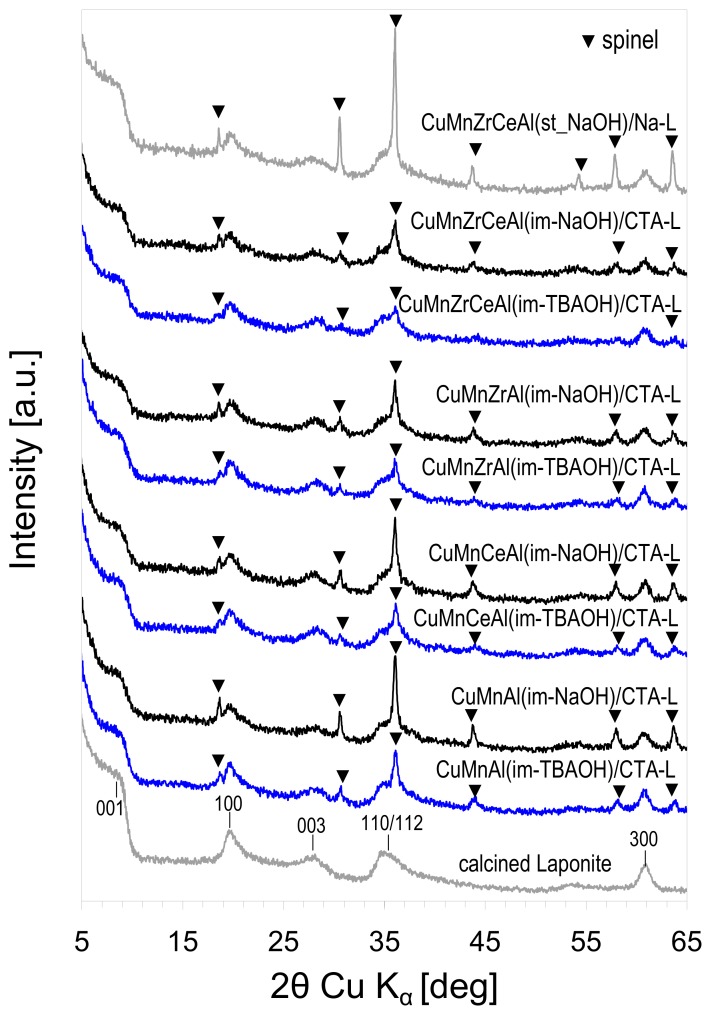
XRD patterns of the investigated composites calcined at 600 °C.

The XRD diagrams of M(1)M(2)…(im_base)/CTA-L composites calcined at 600 °C are shown in Figure 3. The XRD patterns of the reference CuMnAlZrCe(st-NaOH)/Na-L sample and of Laponite calcined at 600 °C are also included. The latter is characterized by the d_001_ value around 1 nm, which is typical of smectite with a dehydrated, collapsed interlayer. The indexing of the calcined Laponite corresponds to the JCPDS 09-0031 reference. Reflections of Laponite can be detected in the XRD patterns of all the investigated samples. In addition, all the composites showed features attributable to a spinel phase of Cu–Mn or a Cu–Mn–Al type (JCPDS files 01-0700262, 01-076-2296, 04-005-7323). For a given composition of the active phase, the spinel reflections were better resolved for materials prepared with NaOH as the precipitant, while for a given precipitating agent, the addition of Zr and/or Ce worsened the spinel pattern resolution. XRD of the reference composite showed that the spinel component derived from Htlc synthesized in a standard manner was by far more crystalline than the composites containing an active phase obtained by the microemulsion method.

**Figure 4:**
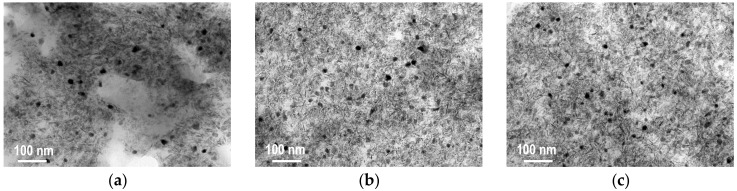
TEM images of (**a**) CuMnAl(im-NaOH)/CTA-L; (**b**) CuMnAl(im-TBAOH)/CTA-L; (**c**) CuMnZrCeAl(im-TBAOH)/CTA-L composites calcined at 600 °C. TBOAH: tetrabutylammonium hydroxide.

TEM images of selected calcined composites showed that pretty uniform grains of active phase (dark spots in various shades of grey) of 20–50-nm size were well intermixed with Laponite, in a manner very similar for all the investigated samples, irrespective of the active phase composition and/or the nature of the precipitating agent (NaOH vs TBAOH) (Figure 4). EDX analysis of selected active phase grains in composites with the most complex mixed oxide composition (CuMnAlZrCe(im-NaOH)/CTA-L and CuMnAlZrCe(im-TBAOH)/CTA-L) showed that all metal elements introduced as precursor components were present in the mixed oxide particles (the Supplementary Materials, Table S1). The Cu/Mn ratio was pretty constant and close to that obtained from XRF analysis, while other elements showed a compositional variation, particularly noticeable for Zr and Ce.

**Figure 5:**
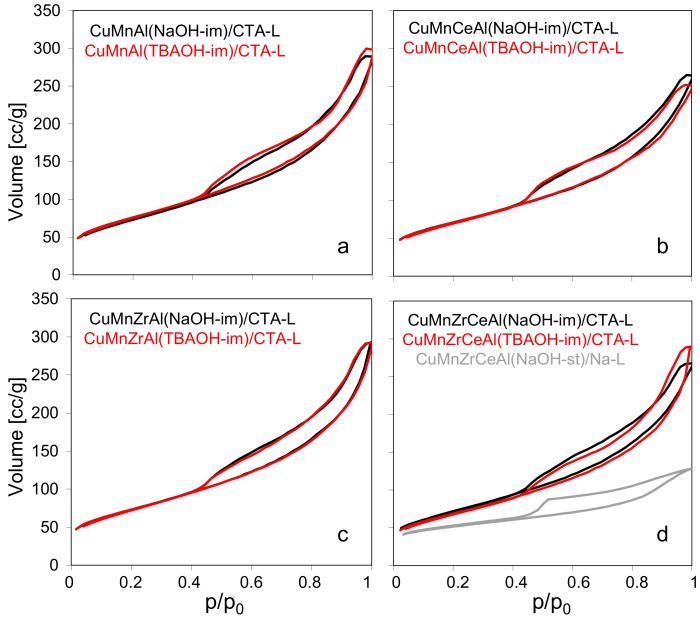
N_2_ adsorption/desorption isotherms of the investigated composites calcined at 600 °C: (**a**) CuMnAl(NaOH)-im)/CTA-L (black) and CuMnAl(TBAOH)-im)/CTA-L (red), (**b**) CuMnCeAl(NaOH)-im)/CTA-L (black) and CuMnCeAl(TBAOH)-im)/CTA-L (red), (**c**) CuMnZrAl(NaOH)-im)/CTA-L (black) and CuMnZrAl(TBAOH)-im)/CTA-L (red), (**d**) CuMnZrCeAl(NaOH)-im)/CTA-L (black), CuMnZrCeAl(TBAOH)-im)/CTA-L (red) and CuMnZrCeAl(NaOH)-st)/Na-L (grey).

All the calcined composite catalysts were subjected to N_2_ adsorption/desorption measurements, and the isotherms recorded at −196 °C are shown in Figure 5. The corresponding textural parameters are presented in Table 2. All the isotherms were of type IV, typical of mesoporous adsorbents, with a characteristic inflection point at relative p/p_0_ close to 1, with H3 hysteresis loops, frequently found in the aggregates of platy particles [30]. It may be seen that both the positions and the shapes of isotherms recorded for the composites with mixed oxide components obtained by micellar synthesis were very similar. As a result, the calculated textural parameters were also very close to each other. The specific surface areas were in the range of 253–273 m^2^/g, the pore volumes ca. 0.39–0.45 cm^3^/g, and the average pore diameters 30.6–34.4 nm. This showed that no matter the type of precipitating agent or the active phase composition, the textural characteristics of the obtained materials were very much alike. Such a result agreed with the TEM analysis of the composites (Figure 4), which revealed that both the size of active phase particles and the manner of their intermixing with Laponite were similar. Moreover, the low values of S_micro_ and V_micro_ confirmed the essentially mesoporous character of the samples obtained by the micellar procedure. In contrast, the isotherm obtained for the reference CuMnZrCeAl(st-NaOH)/Na-L composite, prepared from active phase synthesized by the standard co-precipitation method and the parent Laponite, laid well below all the other isotherms, pointing to the much lower nitrogen uptake capacity, while the data in Table 2 indicated that this material possessed a more pronounced microporosity. The results showed that the synthetic procedure with the use of the microemulsion method for the active phase generation provided materials with better developed textural properties, more suitable for catalytic applications.

**Figure 6:**
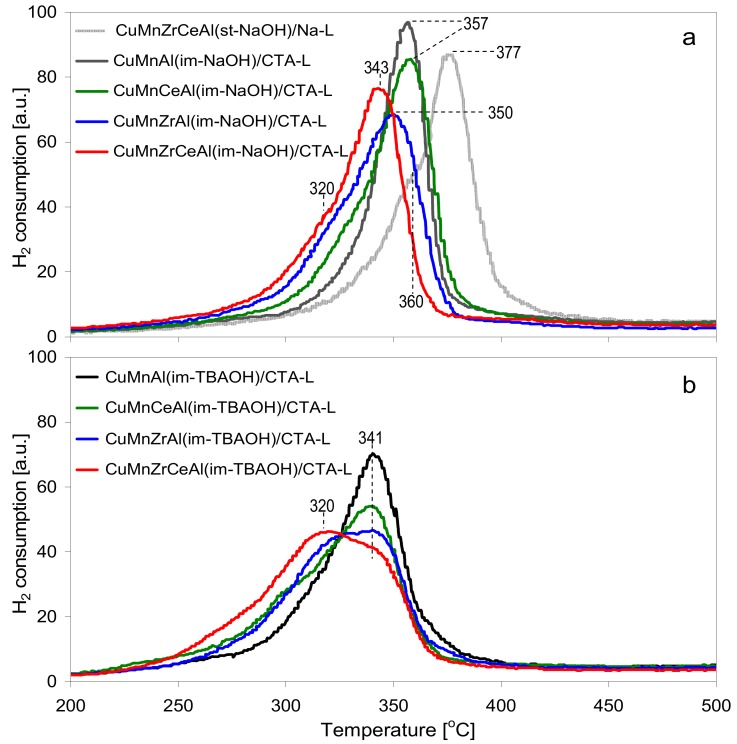
H_2_ TPR profiles of the investigated composites: (**a**) NaOH-precipitated active phase; and (**b**) TBAOH-precipitated active phase.

The catalytic action of transition metal oxides in combustion reactions involves the participation of the catalyst lattice oxygen in the organic molecule transformation (Mars and Van Krevelen mechanism) [31]. To evaluate the ease with which the lattice oxygen was lost from the mixed oxide phase component, temperature-programmed reduction with hydrogen was employed. The H_2_ TPR profiles of all the investigated catalysts are presented in Figure 6. Figure 6a showed the reducibility of the composites whose active phase was precipitated with NaOH, with Figure 6b showing those containing the TBAOH-precipitated active phase. In addition, in Figure 6a, the TPR profile of the reference CuMnZrCeAl(st)/Na-L composite is presented. The TPR profiles in Figure 6a were dominated by a single reduction peak, showing a more or less pronounced asymmetry/shoulder in the direction of low temperature. The TPR curves in Figure 6b were broader and shifted to lower temperature, pointing to the better reducibility of the materials synthesized with aid of TBAOH. Moreover, the low temperature asymmetry/shoulder was generally more pronounced than in the counterpart sample prepared with NaOH. The analysis of both sets of TPR curves showed that the addition of Zr and/or Ce was beneficial for the catalysts’ reducibility, because it increased the contribution of the low-temperature feature and/or shifted the position of the main maximum to lower temperature. It has been repeatedly pointed out that quantitative interpretation of the TPR characteristics of mixed oxides containing both Mn and Cu were quite difficult, due to many factors influencing their reduction, in particular a number of different oxidation states of copper and manganese, which may have been involved in the process [8,13,14,15,16,17,18,19,20,32,33,34,35,36]. In the examined temperature range, Cu^2+^ and/or Cu^+^ underwent reduction to Cu^0^ and Mn^4+^ and/or Mn^3+^ to Mn^2+^. In the present study, the amount of hydrogen consumption attributable to the manganese species (Table 2) was estimated under the assumption that all the copper present in the samples existed as Cu^2+^ and was reduced to Cu^0^ and, in addition, in the Ce-doped samples, cerium transformed from Ce^4+^ to Ce^3+^ [37]. The data in Table 2 showed that under such an assumption one Mn atom accounted for the consumption of ca. 1 H, indicating that the average oxidation state of manganese was about 3+, irrespective of the sample composition and preparation route. The deconvolution of the TPR curves, shown in the Supplementary Materials (Figure S2), revealed that the low-temperature features vary in intensity relative to the high-temperature component. Bearing in mind that the Cu-to-Mn ratio was constant in all samples and that the reduction of copper and manganese cations in the spinel phase is known to occur practically simultaneously [20], it is unlikely that the low-temperature effect was due to the reduction of a particular metal cation. On the other hand, it has been shown that the factor of critical importance in determining the TPR profile of the copper–manganite spinel phase is the degree of crystallinity [15]. In particular, the high-temperature maximum was ascribed to the reduction of a well-ordered copper–manganite spinel, while the low-temperature effect was associated with the reduction of poorly crystalline/amorphous mixed oxide phases. The change in the TPR response of the Cu–Mn–Al-based catalyst upon doping with Zr and Ce was consistent with such an interpretation, as the evolution of the low-temperature TPR feature in the composites was paralleled by the increased amorphization of the mixed oxide phase, as evidenced by the XRD analysis. Similarly, the more amorphous character of mixed oxides generated from the TBAOH-precipitated precursors is believed to account for the better reducibility of the respective composites. In line with this reasoning, the TPR profile of the reference CuMnZrCeAl(NaOH-st)/Na-L sample, in which the spinel phase was most crystalline, was clearly shifted to higher temperature (Figure 6a). It is noteworthy that the addition of Ce and Zr dopants to the TBAOH-precipitated series had a much stronger effect on the reducibility than in the case of the NaOH-synthesized materials. In particular, the deconvolution showed that in the samples containing Ce and/or Zr, besides the two main maxima, a third component, with a maximum below 300 °C, had to be included to account for the most easily reducible species. Its intensity was most pronounced in the catalyst containing both dopants (Figure S1).

**Figure 7:**
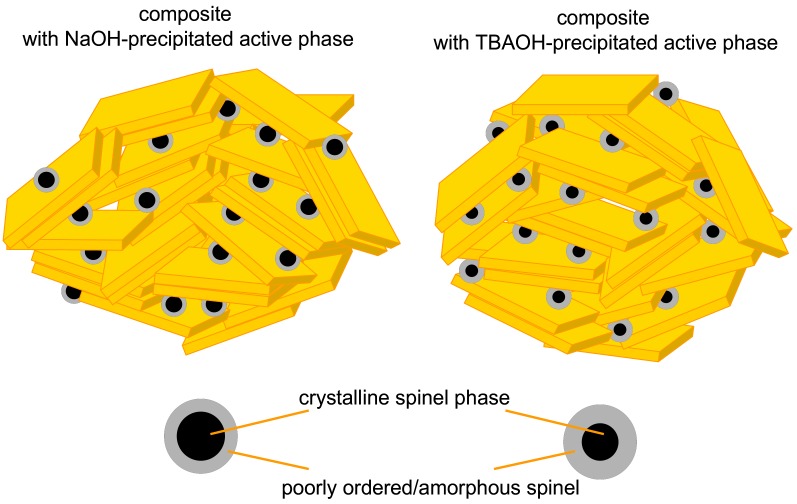
Schematic models of the investigated composite catalysts.

In view of the presented physico-chemical characterization, it follows that the composites of Laponite and the hopcalite-related mixed oxides prepared from inverse microemulsions with the use of two different precipitating bases were mesoporous structures having very similar textural properties in terms of active phase dispersion and intermixing with Laponite layers. However, they differed in the reducibility of the mixed oxide component, with the one formed with use of TBAOH being more susceptible to reduction. The effect was attributed to the higher content of the less ordered/poorly crystalline spinel phase in the materials prepared with TBAOH, as opposed to those synthesized with NaOH. The uniqueness of the nanocrystalline oxide phases obtained from the precursors precipitated with tetraalkylammonium hydroxides, in particular, their low crystallinity, has also been observed by others [38,39]. The schematic models of both types of composites are shown in Figure 7.

### 3.2. Catalytic Testing

**Figure 8:**
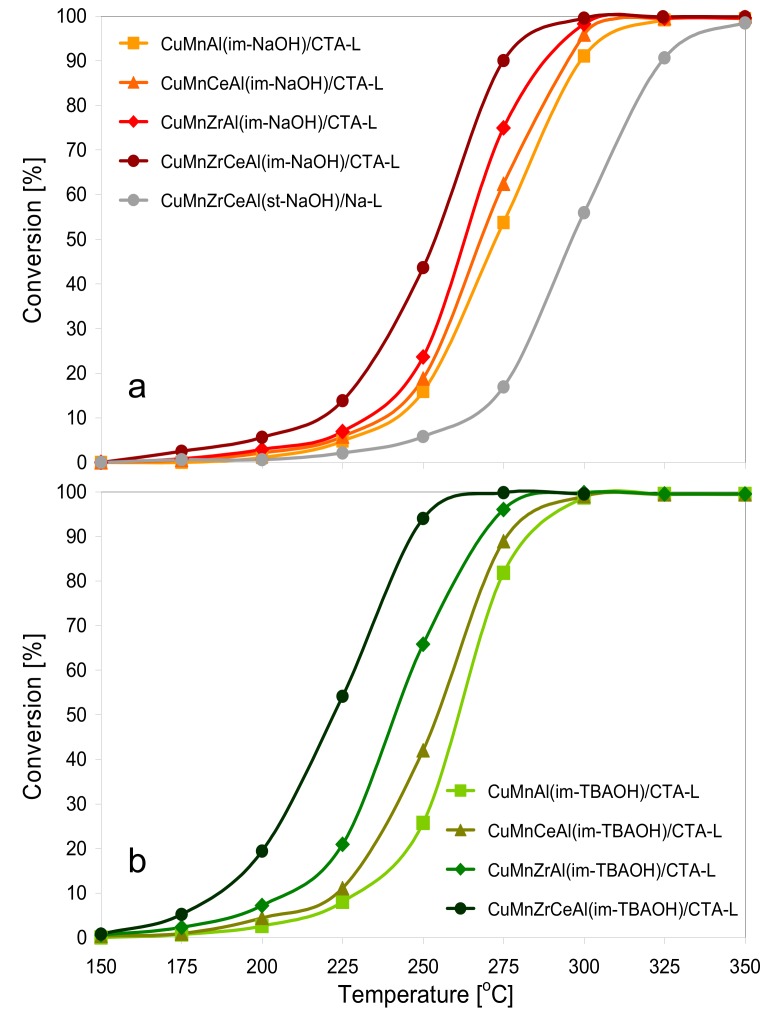
Ignition curves for toluene combustion over the investigated composites calcined at 600 °C: (**a**) the active phase precursor prepared with NaOH; and (**b**) the active phase precursor prepared with TBAOH.

The synthesized composite catalysts were tested in the reaction of total combustion of toluene. The dependence of toluene conversion on reaction temperature is presented in Figure 8a for catalysts whose active phase was synthesized with NaOH and in Figure 8b for those prepared with the use of TBAOH. The T_50_ and T_90_ values (i.e., the temperatures at which 50% and 90% toluene conversion was respectively reached) are shown in Table 2. All the composites containing the active phase obtained by the inverse microemulsion method showed high activity, depending both on the active phase composition and the nature of the precipitating agent. For a given series, the T_50_ and T_90_ temperatures evolved with the active phase composition in a qualitatively similar manner. Thus, within each series, synthesized with either NaOH or TBAOH, the undoped hopcalite-like active phase was the least efficient one, with the performance improving with addition of dopants in the following order: Ce < Zr < Zr + Ce. Remarkably, the spread of the T_50_ and T_90_ values was much more pronounced for the materials synthesized with TBAOH (222–261 and 246–282 °C, respectively) than for the catalysts obtained with the use of NaOH (254–272 and 275–299 °C, respectively). A comparison of Figure 8a with Figure 8b and the data in Table 2 also showed that, in general, the composites whose active phase was prepared with TBAOH were more active than their counterparts obtained with the use of the precursors precipitated with NaOH. Because both series of catalysts displayed pretty similar specific surface areas and porosity, the higher activity of the catalysts synthesized with TBAOH appeared to be caused primarily by the lower crystallinity and, as a consequence, the higher reducibility of the mixed oxides obtained from the TBAOH-precipitated precursors. The results showed that the use of the tetrabutylammonium hydroxide as an unconventional precipitant of the active phase within the inverse micellar system led to composite catalysts of improved combustion activity. In repeated catalytic runs, the catalytic performance of the composites remained unchanged, thus confirming that the prolonged calcination at 600 °C (i.e., at a temperature a great deal higher than that necessary for 100% toluene conversion) ensured stable work under reaction conditions. It should be noted that the reference CuMnZrCeAl(st)/Na-L sample, whose active phase was obtained from the Htlc precursor synthesized by the standard co-precipitation method and was intermixed with parent Laponite, showed a much poorer combustion activity. The effect was attributed to its lower specific surface and pore volume and more crystalline and less reducible copper–manganite spinel phase. Thus, the catalyst design based on combining the active phase prepared by means of the inverse microemulsion method, preferably with the use of TBAOH as the precipitant, with organoclay exfoliated in an organic solvent, was crucial for the preparation of an efficient catalyst.

## 4. Conclusions

Physico-chemical characterization of novel composites of Laponite and hopcalite-related mixed oxides prepared from inverse microemulsions with use of, alternatively, NaOH or TBAOH as precipitating bases, are mesoporous structures with very similar textural properties. However, the copper–manganite nanoparticles formed with the use of TBAOH were less crystalline and, as such, more susceptible to reduction. All the composite catalysts were active in the combustion of toluene, their performance depending on the active phase composition and on the nature of the active phase precipitant. The positive impact of the tested dopants on the performance of the hopcalite-like active phase grows in the following order: Ce < Zr < Zr + Ce. The composites whose active phases were prepared with TBAOH were more active than their counterparts obtained with the use of precursors precipitated with NaOH. The observed patterns of catalytic activity were directly related to the degree of crystalline order and the redox properties of the copper–manganite component: the poorer the crystal order of the spinel phase, the better its reducibility and catalytic properties.

## Figures and Tables

**Table 1:** XRF-determined chemical composition of the composites (non-oxygen elements).

Sample	Si (at.%)	Mg (at.%)	Mn (at.%)	Cu (at.%)	Al (at.%)	Zr (at.%)	Ce (at.%)	Na (at.%)
CuMnAl(im-NaOH)/CTA-L	46.4	31.8	10.9	5.5	5.4	-	-	-
CuMnCeAl(im-NaOH)/CTA-L	46.2	31.3	11.2	5.7	5.2	-	0.4	-
CuMnZrAl(im-NaOH)/CTA-L	46.8	31.6	10.7	5.5	3.3	2.1	-	-
CuMnZrCeAl(im-NaOH)/CTA-L	47.3	31.9	10.6	5.4	2.7	1.8	0.3	-
CuMnAl(im-TBAOH)/CTA-L	47.3	32.2	10.2	5.2	5.1	-	-	-
CuMnCeAl(im-TBAOH)/CTA-L	47.4	32.0	10.1	5.2	5.0	-	0.3	-
CuMnZrAl(im-TBAOH)/CTA-L	46.6	31.9	10.7	5.4	3.2	2.2	-	-
CuMnZrCeAl(im-TBAOH)/CTA-L	47.0	32.1	10.4	5.3	2.8	2.0	0.4	-
CuMnZrCeAl(st)/Na-L	44.5	30.2	11.1	5.6	3.0	2.1	0.4	3.1

**Table 2:** Physicochemical and catalytic characteristics of the composites. S^BET^: specific surface area; V_tot_: total pore volume; S_micro_: micropore specific surface area: V_micro_: micropore volume; D^av^: average pore diameter; H/Mn_TPR_: hydrogen consumption from temperature-programmed reduction (TPR) experiments; T_50_: temperature of 50% toluene conversion; and T_90_: temperature of 90% toluene conversion.

Sample	S_BET_ (m^2^/g)	V_tot_ (cm^3^/g)	S_micro_ (m^2^/g)	V_micro_ (cm^3^/g)	D^av^ (nm)	H/Mn_TPR_	T_50_ (°C)	T_90_ (°C)
CuMnAl(im-NaOH)/CTA-L	265	0.45	14	0.004	33.7	1.0	272	299
CuMnCeAl(im-NaOH)/CTA-L	253	0.41	21	0.008	32.3	1.0	268	295
CuMnZrAl(im-NaOH)/CTA-L	264	0.45	8	0.001	34.3	1.0	263	288
CuMnZrCeAl(im-NaOH)/CTA-L	259	0.41	10	0.002	32.0	0.9	254	275
CuMnAl(im-TBAOH)/CTA-L	273	0.46	13	0.004	33.7	0.9	261	282
CuMnCeAl(im-TBAOH)/CTA-L	254	0.39	15	0.005	30.6	1.1	255	275
CuMnZrAl(im-TBAOH)/CTA-L	262	0.45	16	0.005	34.4	0.9	241	268
CuMnZrCeAl(im-TBAOH)/CTA-L	257	0.45	14	0.004	36.3	1.0	222	246
CuMnZrCeAl(st)/Na-L	189	0.21	62	0.021	44.0	1.0	296	324

## Supplementary Materials

The following are available online at http://www.mdpi.com/1996-1944/11/8/1365/s1, Table S1: EDX-determined atomic ratios of key active phase metal elements to Al in CuMnZrCeAl(im-NaOH)/CTA-L and CuMnZrCeAl(im-TBAOH)/CTA-L.; and Figure S1: Deconvolution of TPR curves.
