# Supplementary material for: Composites of Laponite and Cu–Mn Hopcalite-Related Mixed Oxides Prepared from Inverse Microemulsions as Catalysts for Total Oxidation of Toluene
Source: Materials (Basel). 2018 Aug 6;11(8):1365. doi: 10.3390/ma11081365 (PMC6120024; doi:10.3390/ma11081365)
Supplement: Supplementary file 1 [file materials-11-01365-s001.pdf]

Supplementary Material

Composites of Laponite and Cu–Mn Hopcalite-Related Mixed Oxides Prepared from Inverse Microemulsions as Catalysts for Total Oxidation of Toluene

Bogna D. Napruszewska [1], Alicja Michalik [1], Anna Walczyk [1], Dorota Duraczyńska [1], Roman Dula [1], Wojciech Rojek [1], Lidia Lityńska-Dobrzyńska [2], Krzysztof Bahranowski [3] and Ewa M. Serwicka [1,*]

[1] Jerzy Haber Institute of Catalysis and Surface Chemistry, Niezapominajek 8, 30-239 Krakow, Poland; ncnaprus@cyf-kr.edu.pl (B.D.N.); ncmichal@cyf-kr.edu.pl (A.M.-Z.); ncawalcz@cyf-kr.edu.pl (A.W.); ncduracz@cyf-kr.edu.pl (E.B.); ncdula@cyf-kr.edu.pl (R.D.); ncrojek@cyf-kr.edu.pl (W.R.) and ncserwic@cyf-kr.edu.pl (E.M.S.);

[2] Institute of Metallurgy and Materials Science, Polish Academy of Sciences, Reymonta 25, 30-059 Krakow, Poland; l.litynska@imim.pl (L.L.D.);

[3] AGH University of Science and Technology, Faculty of Geology, Geophysics and Environmental Protection, al. Mickiewicza 30, 30-059 Krakow, Poland; bahr@agh.edu.pl (K.B.);

* Correspondence: ncserwic@cyf-kr.edu.pl; Tel.: +48-12-6395-118

1. Energy Dispersive X-ray (EDX) Measurement

Table S1. EDX-determined atomic ratios of key active phase metal elements to Al in CuMnZrCeAl(im-NaOH)/CTA-L and CuMnZrCeAl(im-TBAOH)/CTA-L. The values obtained from XRF analysis are shown for comparison.

Sample		Cu/Mn	Cu/Zr	Cu/Ce	Cu/Al
CuMnZrCeAl(im-NaOH)/CTA-L	XRF	0.5	3.0	18.0	2.0
	EDS	0.4	4.7	15.1	2.6
		0.5	4.3	11.1	1.9
		0.4	1.3	19.7	1.9
		0.5	2.7	7.7	2.5
CuMnZrCeAl(im-TBAOH)/CTA-L	XRF	0.5	2.7	13.3	1.9
	EDS	0.5	5.2	11.5	1.8
		0.4	2.9	6.7	2.4
		0.5	1.6	11.2	2.5
		0.6	1.7	7.1	1.5

2. Temperature-Programmed Reduction

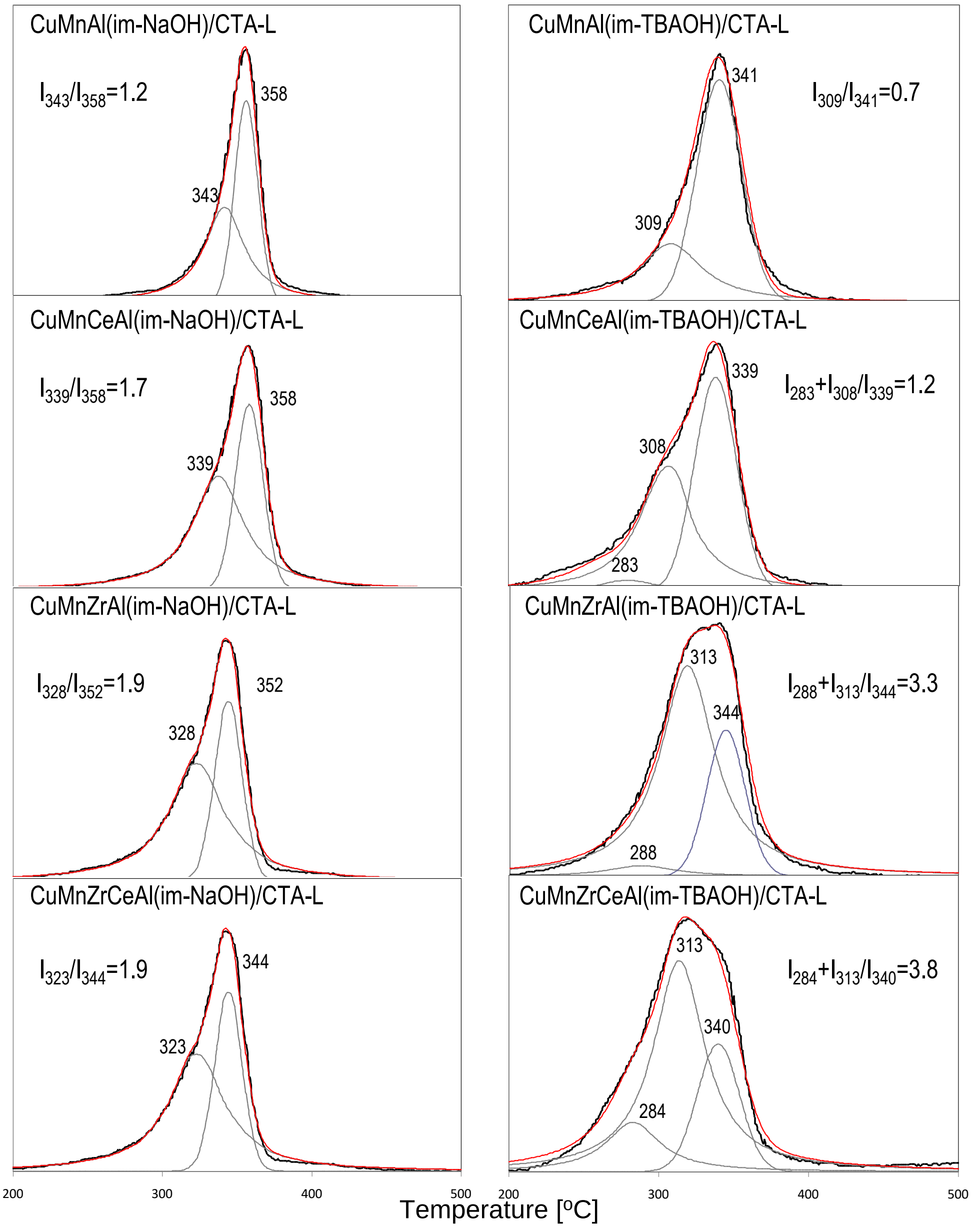

Figure S1. Deconvolution of TPR curves. Original trace: black; simulated: red; and deconvoluted components: grey. Temperatures of component maxima and the ratios of low-temperature to high-temperature effect intensities are given.
